# Genomic profile of extraintestinal pathogenic *Escherichia coli* isolates from prosthetic joint infections: The search for molecular fingerprints

**DOI:** 10.1080/21505594.2026.2613491

**Published:** 2026-01-10

**Authors:** María Ángeles Meléndez-Carmona, M. Carmen Martín-Higuera, Raúl Recio, Eva Benavent, Joan Gómez-Junyent, Mikel Mancheño-Losa, Pilar Hernández-Jiménez, Fernando Chaves, Jaime Lora-Tamayo

**Affiliations:** a Department of Clinical Microbiology, Hospital Universitario 12 de Octubre. Instituto de Investigación Biomédica “i+12” Hospital 12 de Octubre. Madrid, Spain; b Spanish Study Group on Bone and Joint Infections (GEIO-SEIMC); c Department of Infectious Diseases, Hospital Universitario Bellvitge, Barcelona, Spain; d Department of Infectious Diseases. Hospital del Mar. Infectious Pathology and Antimicrobial Research Group (IPAR), CEXS-Universitat Pompeu Fabra, Barcelona, Spain; e Department of Internal Medicine, Hospital Universitario 12 de Octubre. Instituto de Investigación Biomédica “i+12” Hospital 12 de Octubre, Madrid, Spain; f CIBER Enfermedades Infecciosas (CIBERINFEC), Instituto de Salud Carlos III, Madrid, Spain

**Keywords:** Whole-genome-sequencing, biofilm, virulence genes, PJI, antimicrobial resistance

## Abstract

*Escherichia coli* is a key pathogen in extraintestinal infections, including prosthetic joint infections (PJIs), which account for approximately 9% of all such cases. Despite its clinical relevance, the molecular pathogenesis of *E. coli* in PJIs remains poorly defined. This study investigated the clinical, phylogenetic, and virulence profiles of *E. coli* isolates from PJIs and compared them to isolates from bacteremic urinary tract infections (UTIs). A total of 13 isolates from each infection type were analyzed using whole-genome sequencing (WGS) to determine phylogenetic relationships, sequence types, and the presence of virulence genes. PJI isolates exhibited substantial genetic diversity, encompassing 10 sequence types, with ST131 and ST69 being the most frequent. Phylogroup B2 predominated (53.9%) among PJI isolates. Adhesion and biofilm-related genes, such as *fimG/H*, *csg*, and *epaO*, were highly prevalent in PJI isolates, supporting the role of biofilm formation in pathogenesis. Conversely, toxin-associated genes (e.g. *pic* and *senB*) were more frequently detected in UTI isolates. Notably, the *matA* gene, linked to biofilm enhancement, was significantly associated with microbiological failure in PJIs (75% vs. 0%, *p* = 0.02). Phylogenetic analyses revealed no clustering by infection type, suggesting that ExPEC strains share a versatile genomic background, enabling them to adapt to different infection environments. The study highlights the critical role of biofilm formation in PJIs and underscores the genetic adaptability of ExPEC strains, which lack distinct virulence profiles specific to PJIs. However, the small number of PJI isolates limits the generalizability of these findings and warrants confirmation in larger cohorts.

## Introduction

*Escherichia coli* is a highly versatile microorganism with significant genetic diversity that can be systematically classified into various sequence types (STs) and eight phylogroups [1]. While *E. coli* is a key component of the normal commensal gut microbiota in healthy human populations, certain strains possess virulence factors (VFs) that can cause a wide range of intestinal and extraintestinal infections (ExPEC) [2]. Specific ExPEC pathotypes have been identified, such as uropathogenic *E. coli* (UPEC) and neonatal meningitis *E. coli* (NMEC) [3,4]. In contrast to commensal and intestinal pathogenic *E. coli*, ExPEC is primarily derived from phylogroups B2 and D, and specific clones within these groups, which can be identified by their characteristic O:K:H serotypes. Different combinations of virulence factors are associated with these pathotypes. Thus, a single *E. coli* clone has the potential to cause multiple types of infection [5–7].

*E. coli* is also occasionally involved in other extraintestinal infections such as osteomyelitis and prosthetic joint infection (PJI). In the context of the rising incidence of PJI caused by gram-negative bacilli, *E. coli* represents approximately 9% of all PJI cases [8]. However, to our knowledge, no studies have specifically investigated the molecular characteristics of *E. coli* infection in PJI.

To address this gap, the objective of the present study was to analyze by whole-genome-sequencing (WGS) the clinical, phylogenetic relationships, and virulence profile of *E. coli* isolates recovered from patients with PJI, and to compare these isolates with those from bacteremic urinary tract infections (UTIs).

## Material and methods

### Bacterial isolates

A total of 13 *Escherichia coli* isolates that caused PJI were obtained from a prospective, observational, multicenter study conducted at 3 hospitals in Madrid and Barcelona, Spain, between 2019–2020. These are teaching-hospitals with 500–1,000 beds, with multidisciplinary units for the treatment of patients with complex osteoarticular infections. The researchers belong to a common network of clinical research in bone and joint infection (Study Group of Osteoarticular Infection within the Spanish Society of Clinical Microbiology and Infectious Diseases [GEIO-SEIMC]) and share common clinical guidelines on the management of PJI [9].

In addition, 13 *E. coli* strains isolated from patients with bacteremic urinary tract infection (UTI) at one of the hospitals were randomly selected from the blood culture database and retrospectively included as a comparator group. All isolates were stored in cryovials at −80ºC until the experiments were performed.

### Clinical data collection

PJI due to *Escherichia coli* was defined as ≥1 surgical sample, joint aspiration, or blood culture yielding *E. coli*, along with the presence of a typical clinical presentation such as joint pain, erythema and other signs of inflammation, or the presence of a sinus tract or purulence around the prosthesis [10]. PJI was classified as early postsurgical (symptoms of infection beginning within the first 90 days after prosthesis placement), chronic postsurgical (subacute onset of symptoms more than 90 days after prosthesis placement), or hematogenous (acute onset in the setting of suspected bloodstream infection, whether documented or not) [11,12]. Data on clinical presentation (presence of pain, fever, sinus tract, wound dehiscence, wound discharge, plasmatic C-reactive protein, leukocyte blood count), prosthesis characteristics (location, primary or revision device, date of placement), and baseline features (significant comorbidity such as previous diabetes, chronic renal impairment, rheumatoid arthritis, immunosuppression, cirrhosis, chronic heart disease or chronic lung disease) were recorded, as well as data on surgical treatment (type of surgical procedure, date, use of local antibiotics) and the type and duration of antimicrobial therapy. Data was prospectively collected from the clinical charts of patients in an Access database. The study was approved by the Ethics Committee of the Hospital Universitario 12 de Octubre (File 19/145), and patients gave their written informed consent to participate. All patients were adults. Microbiological failure due to initial *E. coli* was used to define death related to the infection and/or the need for salvage therapy or surgery due to the same strain that originally caused the infection. All data was entered into a specially designed Microsoft Access database. All cases were reviewed by three investigators (M.A.M-C, M-M-L and J.L-T) and any discrepancies were verified by the principal investigator at each site.

### Identification and antimicrobial susceptibility testing

The isolation and identification of *E. coli* were conducted in accordance with standard microbiological procedures at each laboratory and then confirmed by MALDI-TOF mass spectrometry and WGS in a central laboratory. Antimicrobial susceptibility testing was performed using the MicroScan Walkaway® System and the E-test method following the European Committee on Antimicrobial Susceptibility Testing (EUCAST) guidelines, version 10.0, 2020 [13].

### Whole-genome sequencing (WGS) and bioinformatics analysis

WGS was performed on all *E. coli* isolates using Nextera XT indexed paired-end libraries from DNA, prepared according to the manufacturer’s instructions and sequenced on a MiSeq platform (Illumina Inc., San Diego, CA, USA) with the MiSeq Reagent Kit v.2 (Illumina Inc.), resulting in 250-bp paired-end reads. The generated raw reads were quality trimmed using the Trimmomatic tool v.0.32 [14] and then de novo assembled using the SPAdes Assembler v.3.11.1 [15]. Genome assembly was evaluated by QUAST v. 5.0.2 [16]. Bacterial identification was confirmed using KmerFinder [17]. The assembled sequences were annotated using Prokka v.1.13.3 [18]. Antimicrobial resistance and virulence genes were identified using the ARIBA [19] with the CARD [20], ResFinder [21] and VFDB and *E. coli* VFDB databases [22]. The assembled genomes were uploaded in FASTA format to the Center for Genomic Epidemiology (CGE) multi-locus sequence typing finder website to identify the sequence types (STs) of the isolates (https://pubmlst.org/bigsdb?db=pubmlst_escherichia_isolates). The Clermont Typer web tool was used for in silico phylogroups: http://clermontyping.iame-research.center/ [23]. To assess the phylogenetic relationship between genomes, non-recombinant core genome SNP analysis was performed using IQ-TREE v.1.6.3 [24] and visualized using the iTOL tool (https://itol.embl.de/).

### Statistical analysis

Categorical variables were described as counts and percentages, whereas continuous variables were expressed as median and interquartile range or mean and standard deviation. Student’s unpaired t-test and the Mann–Whitney U-test were used to compare continuous variables and Fisher’s exact test was used to compare proportions. Only virulence genes with frequencies between 5% and 95% were considered for statistical analysis. All reported *p* values were two-tailed and not adjusted for multiple analysis. The threshold for statistical significance was set at *p* < 0.05. Stata v. 15.1 was used for statistical analysis.

## Results

### Clinical and microbiological characteristics of the PJI cohort

A total of 13 cases of PJI caused by *E. coli* were included. Clinical features and microbiological characteristics are detailed in Table 1. The median age was 70 years (range 52–81) and 30.8% were male. Infection was polymicrobial in three cases (23.1%). Regarding the type of infection, all but one were acute infections: four cases were classified as acute hematogenous PJIs, eight as early post-surgical infections, and one was a chronic post-surgical infection. Eleven patients (84.6%) were managed with debridement, antibiotics and implant retention (DAIR). Median duration of antimicrobials (excluding patients who failed during treatment [*N* = 3]) was 69 days (range 42–91). Six patients (46.2%) were treated mainly with ciprofloxacin for a median of 65 days (range 47–89). Microbiological failure occurred in four cases (30.8%) (three while still under antibiotics, and one after the antimicrobial treatment had been completed), all of which were treated with the DAIR strategy (median follow-up 279 days [IQR:106–800]).

**Table 1.** Clinical and microbiological characteristics of 13 patients with prosthetic joint infection caused by *Escherichia coli*.

**Baseline features**	
Male sex	4 (30.8%)
Age (years) [median (range)]	70.8 (52.5–81.8)
Diabetes	2 (15.4%)
Chronic renal impairment	2 (15.4%)
Rheumatoid arthritis	0 (0%)
Immunosuppressive therapy	1 (7.7%)
Malignancy	0 (0%)
Cirrhosis	0 (0%)
Chronic lung disease	4 (30.8%)
Chronic heart disease	3 (23.1%)
**Clinical presentation**	
Prosthesis location, knee	7 (53.8%)
Hematogenous infection	4 (30.8%)
Time to infection (days)	31.5 (6–60)
Polymicrobial infection	3 (23.1%)
Temperature >37ºC	4 (30.8%)
Sinus tract	1 (7.7%)
Leukocyte count (x 10^9^/L)	9.4 (8.4–13.1)
C- reactive protein (mg/L)	32 (8–85)
**Surgical and antibiotic therapy**	
DAIR	11 (84.6%)
Ciprofloxacin treatment	6 (46.2%)
Need for >1 surgery	6 (46.2%)
Antimicrobial resistance	
Ciprofloxacin	7 (53.8%)
ESBL	2 (18.2%)
**Molecular epidemiology**	
**Clonal complex**	
ST131	3 (23.1%)
ST69	2 (15.4%)
**Others**	8 (61.5%)
**Phylogroup**	
B2	7 (53.9%)
D	2 (15.4%)
A	1 (7.7%)
B1	1 (7.7%)
C	1 (7.7%)
F	1 (7.7%)
**FimH27**	4 (30.8%)
**Microbiological failure**	4 (30.8%)

Abbreviations: DAIR, debridement, antibiotics and implant retention; ESBL, Extended-Spectrum β-lactamases.

^a^Time to infection was calculated for post-surgical infections (early post-surgical infection and chronic post-surgical infection).

^b^The other clonal complexes detected were ST88, ST95, ST127, ST155, ST457, ST1057, ST1193 and ST734.

### Resistance profile of the *E. coli* PJI cohort

Nine strains (69.2%) were resistant to ampicillin. Among them, six strains harbored the TEM-1 gene, a β-lactamase that confers resistance to penicillins and some cephalosporins. Additionally, 2 (15.4%) strains were identified as extended spectrum beta-lactamase (ESBL) producers, harboring the CTX-M-14 and CTX-M-15 genes. Seven strains (53.8%) were resistant to ciprofloxacin, but no mutations were detected in the *qnr* gene. However, these ciprofloxacin-resistant strains carried several genes encoding efflux pumps, which may contribute to the resistance phenotype. All strains were sensitive to carbapenems and did not harbor any genes associated with resistance to these antibiotics.

### Virulence profile of the *E. coli* PJI cohort

**Figure 1.**
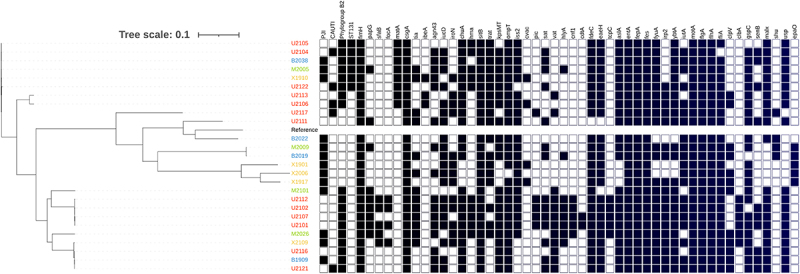
Main molecular characteristics of *Escherichia coli* isolates. Isolates are clustered based on their core genome with the dendrogram displayed on the left. Each row represents one *E. coli* isolate and each column the virulence factor detected. Black squares represent presence of a particular virulence factor, whereas white squares indicate their absence. Only genes found with a frequency of 5–95% were considered of analysis. The isolates were classified based on the type of infection and origin using an alpha numeric code (UTIs- U-XXXX(red); PJI H.U del Mar- M-XXXX (green); H.U bellvitge-B-XXXX (blue); H.U 12 de Octubre X-XXXX (yellow).

The molecular epidemiologic analysis showed that the *E. coli* isolates belonged to 10 different STs, the most frequent being ST131 (23.1%) and ST69 (15.4%). The majority of isolates were assigned to virulence phylogroup B2 (53.9%), followed by phylogroup D (15.4%) (Table 1). The construction of a core genome phylogenetic tree revealed the presence of two clusters with large distances between all PJI *E. coli* isolates, regardless of the hospital of origin (Figure 1).

Overall, the mean number of virulence genes per strain was 211 ± 28.9 in our collection of *E. coli* isolates that caused PJI. The majority of *E. coli* isolates showed a high frequency of adhesin virulence factors associated with biofilm formation. All isolates harbored the adhesin genes *fimG/H*, *csg, fdeC* and *eaeH*, as well as genes related to chemotaxis (*mot*) and motility (*flg*, *flh* and *fli*). Similarly, genes encoding iron uptake factors and protectins were present in most isolates, whereas genes encoding toxins were detected in low frequencies.

Table 2 summarizes the main microbiological characteristics of *E. coli* strains that caused PJI according to clinical presentation (hematogenous vs. postsurgical infection) and clinical outcome. Overall, we did not observe a differential distribution of virulence factors, except in very specific cases. Of note, *E. coli* isolates causing microbiological failure more frequently harbored the *matA* gene (75% versus 0%, *p* = 0.02), a key activator of the *mat* fimbria operon, which enhances *E. coli* biofilm formation.

**Table 2.** Comparison of clinical and genotypic characteristics detected in our *E. coli* PJI cohort.^a^

	All (*N* = 13)	Type of *E. coli* PJI			Outcome		
		Post-surgical infection^b^ (*N* = 9)	Hematogenous infection (*N* = 4)	*P-*value	Success (*N* = 8)	Failure (*N* = 4)^c^	*P*-value
Age (years) median (range)	70.8 (29.2)	68.6 (27.2)	74.4 (19.5)	0.2	70.1 (29.2)	69.1 (17.1)	0.5
Male sex	4 (30.8)	3 (33.3)	1 (25)	1.0	1 (12.5)	2 (50)	0.2
**Underlying conditions**							
Diabetes mellitus	2 (15.4)	1 (11.1)	1 (25)	1.0	2 (25)	0	0.5
Chronic renal impairment	2 (15.4)	0	2 (50)	0.07	2 (25)	0	0.5
Surgical management by DAIR	11 (84.6)	7 (77.8)	4 (100)	1.0	6 (75)	4 (100)	0.5
B2 phylogroup	7 (53.9)	6 (66.7)	1 (25)	0.3	2 (25)	4 (100)	0.06
FimH27	4 (30.8)	2 (22.2)	2 (50)	0.5	4 (50)	0	0.2
**Virulence genes**							
**Adhesins**							
*papG*	4 (30.8)	2 (22.2)	2 (50)	0.5	2 (25)	1 (25)	1.0
*sfaA*	2 (15.4)	2 (22.2)	0	1.0	1 (12.5)	0	1.0
*focA*	1 (7.7)	1 (11.1)	0	1.0	1 (12.5)	0	1.0
*matA*	3 (23.1)	2 (22.2)	1 (25)	1.0	0	3 (75)	**0.02**
**Iron metabolism**							
*iuc*	10 (76.9)	6 (66.7)	4 (100)	0.5	7 (87.5)	3 (75)	1.0
*iroN*	7 (53.9)	5 (55.6)	2 (50)	1.0	5 (62.5)	1 (25)	0.5
*chuA*	10 (76.9)	7 (77.8)	3 (75)	1.0	5 (62.5)	4 (100)	0.5
*fyu*	10 (76.9)	6 (66.7)	4 (100)	0.5	5 (62.5)	4 (100)	0.5
*irp*	7 (53.9)	4 (44.4)	3 (75)	0.6	5 (62.5)	1 (25)	0.5
*ybt*	10 (76.9)	6 (66.7)	4 (100)	0.5	5 (62.5)	4 (100)	0.5
*iutA*	10 (76.9)	6 (66.7)	4 (100)	0.5	7 (87.5)	3 (75)	1.0
**Protectins**							
*traT*	9 (69.2)	6 (66.7)	3 (75)	1.0	5 (62.5)	3 (75)	1.0
*kpsM*	10 (76.9)	7 (77.8)	3 (75)	1.0	5 (62.5)	4 (100)	1.0
*ompA*	12 (92.3)	8 (88.9)	4 (100)	1.0	7 (87.5)	4 (100)	1.0
*iss*	11 (84.6)	7 (77.8)	4 (100)	1.0	7 (87.5)	3 (75)	1.0
*cvaC*	3 (23.1)	2 (22.2)	1 (25)	1.0	2 (25)	1 (25)	1.0
**Toxins**							
*sat*	6 (46.2)	3 (33.3)	3 (75)	0.3	3 (37.5)	3 (75)	0.5
*vat*	4 (30.8)	4 (44.4)	0	0.2	2 (25)	1 (25)	1.0
*hlyA*	4 (30.8)	2 (22.2)	2 (50)	0.5	2 (25)	1 (25)	1.0
*senB*	2 (15.4)	2 (22.2)	0	1.0	0	2 (50)	0.09
*clb*	2 (15.4)	2 (22.2)	0	1.0	1 (12.5)	0	1.0
*usp*	7 (53.9)	6 (66.7)	1 (25)	0.3	2 (25)	4 (100)	0.06
**Secretion system**							
*clpV*	8 (61.5)	5 (55.6)	3 (75)	1.0	6 (75)	1 (25)	0.2
*gspC*	12 (92.3)	9 (100)	3 (75)	0.3	7 (87.5)	4 (100)	1.0
**Pathogenicity Island**							
*malX*	8 (61.5)	7 (77.8)	1 (25)	0.2	3 (37.5)	4 (100)	0.08

^a^Only genes with frequencies ranging from 5% to 95% are included in the table.

^b^There were eight early post-surgical infections and one chronic post-surgical infection.

^c^One case was excluded from outcome analysis due to death unrelated to the infection.

### Genomic comparison of *E. coli* isolated from PJI versus UTI

Thirteen *E. coli* isolates causing bacteremic UTI were included. The median age of patients was 78.7 years (range 74.4–86.7) and 61.5% were male. Four of these isolates (30.8%) were catheter-associated urinary tract infections. Antimicrobial resistance profile of these strains is resumed in Supplementary Table 1.

Figure 1 shows the main microbiological characteristics of the two groups. As with the PJI strains, most of the *E. coli* UTI isolates belonged to phylogroup B2 (84.6%). However, the dominant sequence type among UTI isolates was ST73 (30.8%) followed by ST131 (23.1%). The phylogenetic tree of *E. coli* isolates did not show clustering by type of infection (Figure 1).

With respect to the virulence genes, we did not observe a signifcanltly higher number of virulence genes in the PJI group compared to the UTI group (211 ± 28.9 versus 205.6 ± 26.8, *p* = 0.7). Thus, the virulence profile was very similar. However, the PJI-causing *E. coli* isolates compared to those causing UTIs more frequently carried the *epaO* gene (38.5% versus 0%, respectively; *p* = 0.04), whereas the UTI isolates more frequently carried toxin genes *pic* (38.5% versus 0%, *p* = 0.04), *senB* (61.5% versus 15.4%, *p* = 0.04), and the pathogenicity island marker *malX* (100% versus 61.5%; *p* = 0.04) (Table 3). A new comparative analysis between PJI- and UTI-causing *E. coli* was performed after excluding catheter-associated strains and similar results were found (data not shown). We also analyzed potential differences in virulence profiles between the 4 cases of hematogenous PJI and the 13 cases from bacteremic UTI. However, we found a very similar virulence pattern between the two groups, with no significant differences observed. Finally, we also investigated the presence of specific gene associations previously described in ExPEC as possible genomic markers of *E. coli* PJI isolates and found no greater presence of any gene association in the PJI group compared to the UTI control group (Table 4).

**Table 3.** Comparison of clinical and genotypic characteristics detected in our *E. coli* PJI versus *E. coli* UTI^a^.

	All (*N* = 26)	Type of infection		
		*E. coli* PJI (*N* = 13)	*E. coli* UTI (*N* = 13)	*P-*value
Age (years), median (range)	76.8 (43.8)	70.8 (29.2)	78.7 (39.9)	**0.02**
Male sex	12 (46.2)	4 (30.8)	8 (61.5)	0.2
B2 phylogroup	18 (69.2)	7 (53.9)	11 (84.6)	0.2
FimH27	4 (15.4)	4 (30.8)	0 (0)	0.09
**Virulence genes**				
**Adhesins**				
*papG*	9 (34.6)	4 (30.8)	5 (38.5)	1.0
*sfaA*	5 (19.2)	2 (15.4)	3 (23.1)	1.0
*focA*	4 (15.4)	1 (7.7)	3 (23.1)	0.6
*matA*	8 (30.8)	3 (23.1)	5 (38.5)	0.7
***Iron metabolism***				
*iuc*	20 (76.9)	10 (76.9)	10 (76.9)	1.0
*iroN*	13 (50)	7 (53.9)	6 (46.2)	1.0
*chuA*	23 (88.5)	10 (76.9)	13 (100)	0.2
*fyu*	23 (88.5)	10 (76.9)	13 (100)	0.2
*irp*	18 (69.2)	7 (53.9)	11 (84.6)	0.2
*ybt*	23 (88.5)	10 (76.9)	13 (100)	0.2
*iutA*	20 (76.9)	10 (76.9)	10 (76.9)	1.0
**Protectins**				
*traT*	18 (69.2)	9 (69.2)	9 (69.2)	1.0
*kpsM*	21 (80.8)	10 (76.9)	11 (84.6)	1.0
*iss*	21 (80.8)	11 (84.6)	10 (76.9)	1.0
*cvaC*	4 (15.4)	3 (23.1)	1 (7.7)	0.6
*epaO*	5 (19.2)	5 (38.5)	0 (0)	**0.04**
**Toxins**				
*sat*	14 (53.9)	6 (46.2)	8 (61.5)	0.7
*vat*	12 (46.2)	4 (30.8)	8 (61.5)	0.2
*hlyA*	8 (30.8)	4 (30.8)	4 (30.8)	1.0
*senB*	10 (38.5)	2 (15.4)	8 (61.5)	**0.04**
*clb*	6 (23.1)	2 (15.4)	4 (30.8)	0.6
*usp*	19 (73.1)	7 (53.9)	12 (92.3)	0.07
*shu*	4 (15.4)	3 (23.1)	1 (7.7)	0.6
*pic*	5 (19.2)	0 (0)	5 (38.5)	**0.04**
*cnf1*	5 (19.2)	1 (7.7)	4 (30.8)	0.3
**Secretion system**				
*clpV*	12 (46.2)	8 (61.5)	4 (30.8)	0.2
*gspC*	23 (88.5)	12 (92.3)	11 (84.6)	1.0
**Pathogenicity Island**				
*malX*	21 (80.77)	8 (61.5)	13 (100)	**0.04**

Abbreviations: PJI, prosthetic joint infection; UTI, urinary tract infection.^a^Only genes with frequencies ranging from 5% to 95% are included in the table.

**Table 4.** Examination of the co-occurrence of two virulence factor-encoding genes in the studied *E. coli* isolates.

Combination of VF	*E. coli* PJI isolates		*E. coli* UTI isolates		*P*
	n	%	n	%	
*hlyA+iutA*	3	23.1	4	30.8	1.0
*agn43+papC*	3	23.1	5	38.5	0.7
*kspMT/agn43*	6	46.2	9	69.2	0.4
*usp+papC*	3	23.1	5	38.5	0.7
*iutA+agn43*	5	38.5	8	61.5	0.4

VF: virulence factors.

## Discussion

Our study highlights the complexity and diversity of *Escherichia coli* isolates that cause PJI. Despite a common clinical context, the isolates displayed a wide genetic diversity across different sequence types and phylogroups, and exhibited a wide array of virulence factors, particularly those associated with biofilm formation and adhesion.

Molecular typing revealed that PJI isolates belonged to 10 different sequence types (STs), with ST131 and ST69 being the most prevalent, and B2 being the predominant phylogroup. The high prevalence of the B2 phylogroup in our PJI isolates is consistent with studies on ExPEC including *E. coli* strains that cause urinary tract and bloodstream infections, where B2 strains are known for their enhanced virulence potential, including the ability to form biofilms and evade host immune responses [3,25–27]. In line with previous studies, we observed an association between PJI isolates belonging to the ST131 lineage and ciprofloxacin resistance, which underscores the clinical relevance of monitoring antimicrobial resistance patterns in ST131-related infections [28].

The high presence of adhesins and biofilm-associated genes, such as *fimG/H* and *csg*, is also consistent with other studies on *E. coli* associated with medical device-related infections [29]. Biofilm formation is a major virulence mechanism in PJI, facilitating bacterial persistence and resistance to both immune responses and antibiotic treatment [30]. The widespread presence of iron acquisition systems and protectins further corroborates the role that these factors play in enhancing bacterial survival in nutrient-limited environments, such as those found at infection sites [31].

However, in contrast to some studies that have reported a higher prevalence of toxin-encoding genes such as *hlyA* and *cnf1* in ExPEC isolates [32], we observed a lower frequency of these virulence determinants in our PJI collection. This suggests that toxin production may play a lesser role in the pathogenesis of orthopedic implant infections than in other infection types where tissue damage and immunomodulation would be more critical. The association between the *matA* gene and microbiological failure in our study emphasizes the importance of biofilm-related factors in the persistence of PJI caused by *E. coli* and treatment failure [33]. This is consistent with other research showing that increased biofilm formation contributes to recalcitrant chronic infections and complicates antimicrobial therapy [30].

The genomic comparison of *E. coli* isolates from PJIs and UTIs provides important insights into the virulence factors and phylogenetic relationships among these pathogenic strains. We decided to use strains causing UTI as a comparator group, since these are the most common extra-intestinal infections caused by *E. coli*. Apart from the predominance of phylogroup B2 in both types of infection, we observed heterogeneity and a diverse virulence gene profile among all strains analyzed, with no specific gene aggregation by PJI or UTI. In our analysis, no statistically significant differences in the mean number of virulence genes were found between the PJI and UTI groups, indicating a remarkably similar profile in terms of virulence and load. However, there were notable differences in specific virulence genes. The *epaO* gene, which has been implicated in biofilm formation and adherence in *Enterococcus faecalis* [34], was significantly more frequent in PJI isolates. This suggests that biofilm formation could play a critical role in the pathogenicity of *E. coli* in PJI, where adherence to prosthetic devices is a key factor in the establishment of infection. Conversely, UTI isolates exhibited a higher prevalence of toxin-associated genes such as *pic* and *senB*. This underscores the differential virulence mechanisms employed by *E. coli* in UTI, where toxins contribute to mucosal damage and inflammation.

Our univariate analysis to identify potential gene associations previously described in ExPEC did not reveal significant differences between the two groups [35]. This raises important questions regarding the pathogenic potential of *E. coli* strains with no specific virulence gene profile. The finding that no single virulence gene or combination of genes consistently correlates with type of infection suggests that the pathogenic capacity of ExPEC strains is more broadly based. It is reasonable to suggest that any *E. coli* isolate with the right genomic background including adherence factors and potential resistance mechanisms, can produce various types of infection without being constrained to a fixed pattern of virulence genes. This adaptability is consistent with the idea of ExPEC as harboring a heterogeneous group of genes capable of causing a wide spectrum of infections, ultimately depending on host factors and environmental conditions.

This study has several limitations that should be taken into account when interpreting the results. First, the sample size is relatively small. The small number of cases may reduce the statistical power of the analyses and limit the generalizability of our findings to broader populations. This is particularly relevant given the rarity of *E. coli*-associated PJIs, which makes larger, multicentric datasets necessary to validate our observations. Therefore, caution is advised when extrapolating these results to other settings. A larger cohort would provide a more robust dataset to detect subtle differences in virulence gene profiles and phylogenetic relationships. In addition, the inclusion of polymicrobial cases may have introduced some bias regarding the comparisons of strains based on clinical outcomes. Second, the study focused primarily on specific virulence genes and did not explore other possible genetic factors such as mobile genetic elements or regulatory sequences that may also contribute to the pathogenicity of *E. coli*. Third, the comparison was made exclusively with a cohort of urinary tract infections and did not include other types of extraintestinal infections. We chose a 1:1 comparison, but the inclusion of a larger number of ITU strains could have increased the statistical power of our analysis. Moreover, urinary strains were recovered from one center instead of the three participating centers. Fourth, the presence of certain genes does not imply their expression. It would have been very informative if the study had been able to correlate with the expression of genes rather than simply their presence or absence. In future follow-up studies, it would be beneficial to conduct phenotypic biofilm quantification to validate the genomic findings, particularly given the association observed between biofilm-related genes and microbiological failure. Finally, we performed multiple statistical comparisons that may have led to spurious associations between the presence of certain genes in specific groups of *E. coli* strains. Nevertheless, this would have reinforced one of our major findings, which was that there were no significant genotypic differences between the strains of ExPEC studied, regardless of the specific clinical syndrome caused.

In conclusion, our study illustrates the significant genetic diversity of *Escherichia coli* strains involved in PJIs, highlighting the critical role of biofilm formation in their pathogenicity. The absence of a specific virulence pattern associated with distinct pathotypes suggests that any *E. coli* isolate can cause a variety of extraintestinal infections, including PJI. Although PJIs due to *E. coli* are relatively rare, their clinical impact given the complexity of management and the risk of treatment failure makes it essential to better understand the molecular characteristics of the strains involved. These findings underline the importance of understanding the adaptability of *E. coli* and open up new avenues for investigating the genetic determinants of pathogenicity and the interplay between host factors and bacterial characteristics. Such knowledge may ultimately lead to more effective strategies for the prevention and treatment of *E. coli*-related infections.

## Supplementary Material

Clean Copy of Supplementary Table- QVIR-2025-0015.R1.docx

## Data Availability

Sequence files were deposited at GenBank under BioProject PRJNA1191230 (https://www.ncbi.nlm.nih.gov/bioproject/?term=PRJNA1191230) and accession numbers SAMN45074931-SAMN45074956.
