# Supplementary material for: Genomic profile of extraintestinal pathogenic Escherichia coli isolates from prosthetic joint infections: The search for molecular fingerprints
Source: Virulence. 2026 Jan 10;17(1):2613491. doi: 10.1080/21505594.2026.2613491 (PMC12795266; doi:10.1080/21505594.2026.2613491)
Supplement: Clean Copy of Supplementary Table- QVIR-2025-0015.R1.docx [file KVIR_A_2613491_SM8374.docx]

**Supplementary Table 1 – Antimicrobial susceptibility of the urinary strains used in the study**

| **Strain** | **AMP** | **AMC** | **PTZ** | **CRO** | **GEN** | **ERT** | **MER** | **CIP** | **CMX** |
| --- | --- | --- | --- | --- | --- | --- | --- | --- | --- |
| U2102 | R | S | S | S | S | S | S | S | S |
| U2105 | R | S | S | R | S | S | S | R | R |
| U2107 | S | S | S | S | S | S | S | S | S |
| U2111 | S | S | S | S | S | S | S | S | S |
| U2112 | R | S | S | S | S | S | S | S | S |
| U2113 | S | S | S | S | S | S | S | S | S |
| U2116 | R | R | S | S | S | S | S | S | S |
| U2104 | R | S | S | R | S | S | S | R | S |
| U2106 | S | S | S | S | S | S | S | S | S |
| U2117 | R | R | R | R | I | S | S | R | R |
| U2121 | S | S | S | S | S | S | S | R | S |
| U2122 | R | S | S | S | S | S | S | S | S |
| U2101 | R | R | S | S | S | S | S | S | S |

Antimicrobial susceptibility was analyzed by automated microdilution (MicroScan Walkaway® System, Siemens, California, EE. UU). AMP: ampicillin; AMC: amoxicillin/clavulanate; PTZ: piperacillin/tazobactam; GEN: gentamicin; ERT: ertapenem; MER: meropenem; CIP: ciprofloxacin; CMX: co-trimoxazole. Antimicrobial susceptibility according to EUCAST, S: susceptible; I: intermediate; R: resistant.
